# Dynamic Compressive Mechanical Behavior of a Novel Three-Dimensional Re-Entrant Honeycomb (3D-RH) Structure

**DOI:** 10.3390/ma18225234

**Published:** 2025-11-19

**Authors:** Xiyan Du, Lun Qi, Yulong Shi, Lei Xing, Gang Wang, Haibo Zhang, Wenting Bai, Xiaofei Cao, Chunwang He

**Affiliations:** 1 China Nuclear Power Engineering Co., Ltd., Hebei Branch, Shijiazhuang 050021, China; 2 School of Urban Geology and Engineering, Hebei GEO University, Shijiazhuang 050031, China; 3 Hubei Key Laboratory of Theory and Application of Advanced Materials Mechanics, Department of Engineering Mechanics, School of Physics and Mechanics, Wuhan University of Technology, Wuhan 430070, China; 4 Institute of Advanced Structure Technology, Beijing Institute of Technology, Beijing 100081, China

**Keywords:** mechanical behavior, dynamic compression, three-dimensional re-entrant honeycomb, mechanical metamaterials

## Abstract

Negative Poisson’s ratio structural materials have unique deformation characteristics and excellent mechanical properties, and are widely used in multiple key fields, such as aerospace, nuclear safety, rail transit, and so on. However, most of them are two-dimensional negative Poisson’s ratio structural materials, and the mechanical design and performance evaluation of dynamic behavior of three-dimensional novel negative Poisson’s ratio structural materials deserve more attention. Inspired by the deformation mechanism of the traditional two-dimensional re-entrant honeycomb (2D-RH) structure, this study extends the planar structural characteristics to the spatial dimension and proposes a novel three-dimensional re-entrant honeycomb (3D-RH) structure. Experimental testing, theoretical analysis, and numerical simulation are all utilized to study its quasi-static and dynamic compressive mechanical properties and deformation processes. The novelty of this paper lies in the novel 3D-RH structure and the investigation of the static and dynamic mechanical behavior. The testing results indicate that the quasi-static compressive performance curve of the 3D-RH pattern is a typical bending-dominated deformation behavior, and the dynamic mechanical properties of the 3D-RH structural pattern exhibit an apparent strain rate effect. In addition, Ashby maps are also plotted to demonstrate its acceptable performance characteristics, indicating its potential attractive application prospects in innovative development of lightweight, high-specific-stiffness, and high-specific-strength structural materials.

## 1. Introduction

Mechanical metamaterials are a class of artificial structural materials with specific microstructures, mesoscopic connections, and macroscopic layouts [1,2,3]. They have various advantages, such as high performance and multifunctionality, reconfigurability and programmability, sound isolation, thermal insulation, and heat transfer, which have shown great potential and industrial value in many key engineering fields such as aerospace, nuclear safety, construction engineering, rail transit, and biomedicine [4,5,6,7,8,9]. Among them, negative Poisson’s ratio structural materials [10] are a typical type of mechanical metamaterials. Owing to their unique microstructure and deformation mechanism, they exhibit completely different mechanical behaviors from ordinary materials when subjected to external loads. This characteristic endows them with a stronger load-bearing capacity and higher energy absorption properties, resulting in better fatigue resistance and impact resistance. There are various types of negative Poisson’s ratio structural materials, among which re-entrant honeycomb structural materials [11] have gradually attracted widespread attention and in-depth research from researchers due to their unique design methods, concise load-bearing paths, and clear deformation mechanisms [12,13,14].

In terms of the mechanical design method, traditional re-entrant honeycomb design strategies mainly include gradient design [15], hierarchical design [16], curved ligament design [17], hybrid design [18], biomimetic design [19], and so on [20]. Researchers have developed different design methods to improve the mechanical performance and achieve goals such as light weights, high strengths, high energy absorption efficiencies, and specific physical properties (such as a negative Poisson’s ratio). However, it should be noted that most of the designs for re-entrant honeycombs are on a two-dimensional scale. Three-dimensional designs are still limited, especially the creative design of the 3D-RH pattern inspired by the deformation mechanism of the 2D-RH pattern. It is an innovative attempt to extend the characteristics of planar structures to spatial dimensions which deserves more attention.

As for the performance evaluation of 2D-RH patterns, Deng [21] proposed a novel type of re-entrant honeycomb and investigated its in-plane impact performance and energy absorption characteristics numerically. Chen [22] proposed a re-entrant chiral honeycomb with an enhanced orthogonal anisotropy. Theoretical analysis, experimental tests, and numerical simulations are all adopted to study its quasi-static mechanical behavior. Chen [23] proposed a novel auxetic honeycomb structure enhanced with self-similar inclusion, and its superior quasi-static performance characteristics have been demonstrated compared to the conventional re-entrant hexagonal honeycomb. Zhang [24] designed a novel re-entrant double-arrow honeycomb and studied its quasi-static and dynamic crushing behaviors in different loading directions. Most researchers focus on exploring the elastic and plastic mechanical properties of 2D-RH structural materials under quasi-static loading conditions, analyzing their deformation processes and revealing their failure mechanisms.

In recent years, researchers have made some attempts to evaluate the mechanical properties of 3D-RH patterns. Wang [25] proposed a novel periodic auxetic reentrant honeycomb structure assembled with a new octagon honeycomb unit cell. The corresponding mechanical performance of the proposed honeycomb structure was experimentally and numerically investigated under quasi-static compressive loading. Shao [26] proposed a 3D re-entrant anti-trichiral honeycomb structure, and its quasi-static compression behavior, auxeticity, and energy absorption capabilities were investigated through numerical simulations and experimental tests. Zhang [27] designed two 3D honeycombs, and their three-point bending behavior was studied. The testing results indicated that the 3D auxetic re-entrant honeycomb exhibited a higher ductility and fracture resistance compared with the 3D non-auxetic hexagonal honeycomb. Although some attempts have been made to study the mechanical behavior of 3D-RH patterns, more attention still needs to be paid, especially to the dynamic characteristics of 3D-RH structural patterns.

Thus, inspired by the deformation mechanism of the traditional two-dimensional re-entrant honeycomb (2D-RH) structure, this study extends the planar structural characteristics to the spatial dimension and proposes a novel three-dimensional re-entrant honeycomb (3D-RH) structure. A series of methods including experimental testing, theoretical analysis, and numerical simulation are all utilized to study its quasi-static and dynamic compressive mechanical properties and deformation processes. The novelty of this paper lies in the novel 3D-RH structure and the investigation of the static and dynamic mechanical behavior. Ashby maps are also plotted to demonstrate its acceptable performance characteristics, indicating its potential attractive application prospects in the innovative development of lightweight, high-specific-stiffness, and high-specific-strength structural materials. This paper will provide valuable guidance for the mechanical design and performance evaluation of the novel three-dimensional negative Poisson’s ratio structural materials.

## 2. Methods and Models

In this section, the architecture design, theoretical analysis, and computational model of the 3D-RH structure are presented. Firstly, the architecture design of the 3D-RH structure, including the design process and geometric parameters, are discussed. Then, theoretical analyses of the equivalent compressive modulus and compressive strength are described. Finally, detailed settings with regard to the quasi-static and dynamic compression computational model, including the boundary conditions, contact types, material parameters, mesh division, and mesh sensitivity, are all clarified.

### 2.1. Architecture Design

**Figure 1:**
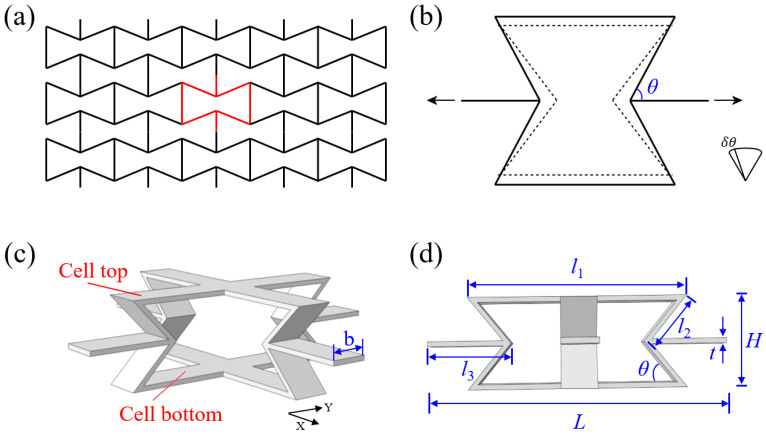
(**a**) Structural configuration and (**b**) deformation diagram of the traditional two-dimensional re-entrant honeycomb (2D-RH) structure; (**c**) three-dimensional re-entrant honeycomb (3D-RH) structure and the corresponding (**d**) geometric parameters.

Figure 1a illustrates the structural configuration of the traditional two-dimensional re-entrant honeycomb (2D-RH) structure, and the corresponding deformation diagram is shown in Figure 1b. Its spatial structure can be obtained by stretching the cells along the out-of-plane direction for a certain distance, and the geometric topology shape of the cells determines the auxetic characteristics of the structure. When the structure is subjected under a vertical tensile load, its oblique cell walls will rotate outward around the hinge node, driving adjacent cells to translate outward, thereby causing the structure to expand and exhibiting a negative Poisson’s ratio effect.

Inspired by the deformation mechanism of the 2D-RH structure, a novel three-dimensional re-entrant honeycomb (3D-RH) structure is constructed by expanding the dimensions of the two-dimensional structure. The 3D-RH structure exhibits negative Poisson’s ratio effects in both X and Y directions, making it more pronounced than the traditional 2D-RH structure. Thus, it can be inferred that it has a higher compressive strength and superior energy absorption properties. As shown in Figure 1c, the 3D-RH cell is generated by two identical 2D-RH cells that are orthogonal to each other, and the final 3D-RH structure can be formed by arranging along three main directions. Detailed geometric parameters are marked in Figure 1d, including the horizontal cell wall length $l_{1}$, oblique cell wall length $l_{2}$, connecting cell wall length $l_{3}$, cell wall width b, cell wall thickness t, and re-entrant angle θ. All the geometric parameter values are listed in Table 1. Then, based on the above geometric parameters, the unit cell length L and unit cell height H can, respectively, be expressed as follows:

(1) $$\left\{\begin{array}{l} H=2l_{2}\sin\theta \\ L=l_{1}-2l_{2}\cos\theta +2l_{3} \end{array}\right.$$

### 2.2. Theoretical Analysis

**Figure 2:**
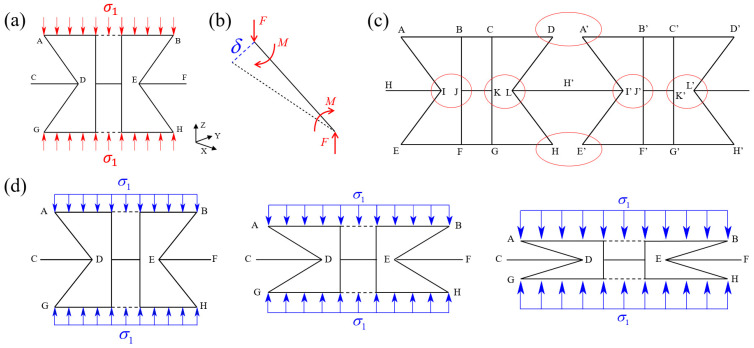
Force diagram of the (**a**) unit cell and (**b**) oblique cell wall; (**c**) the simplified diagram of 3D-RH adjacent cells; (**d**) schematic diagram of the 3D-RH pattern under quasi-static compression.

In this section, theoretical analyses of the equivalent compressive modulus and compressive strength of the 3D-RH pattern are conducted. In the initial stage of quasi-static compression, deformation of the 3D-RH cell is mainly dominated by the elastic bending of the oblique cell walls, and the whole structure is in the stage of elastic deformation. Each oblique cell wall can be simplified as a beam with thickness of t, width of b, and elastic modulus of E. The Euler–Bernoulli beam assumption and small deformation assumption are considered. Based on the Euler–Bernoulli beam theory, the cross-section of the beam does not change during bending, and the shear deformation and axial deformation are both neglected. As shown in Figure 2a, the load is symmetrically distributed along the x-z plane or y-z plane at the center of the structure. According to the force balance condition, the force on the cell in the horizontal direction is zero. Thus, the oblique cell wall AD is extracted for force analysis, and the force diagram is shown in Figure 2b.

The equivalent external force F and equivalent bending moment M at both ends of the oblique cell wall can be, respectively, expressed as follows:

(2) $$F=\frac{\sigma_{1}b(2l_{1}-b)}{4}$$

(3) $$M=\frac{1}{2}Fl_{2}\cos\theta$$

Then, the displacement in the vertical direction is as follows:

(4) $$\delta \cos\theta =\left(\frac{Fl_{2}^{3}\cos\theta }{3EI}-\frac{Ml_{2}^{2}}{2EI}\right)\cos\theta =\frac{Fl_{2}^{3}\cos^{2}\theta }{12EI}$$

where $I=bt^{3}/12$ is the inertia moment of the cross-section of the oblique cell wall.

The strain in the vertical direction is as follows:

(5) $$\varepsilon_{z}=\frac{\delta \cos\theta }{l_{2}\sin\theta }$$

Finally, the equivalent modulus $E_{z}$ in the compression direction is as follows:

(6) $$E_{z}=\frac{\sigma_{1}}{\varepsilon_{z}}=\frac{4Et^{3}\sin\theta }{(2l_{1}-b)l_{2}^{2}\cos^{2}\theta }$$

Herein, one should note that, in order to prevent the theoretical solution deviation of mechanical parameters caused by mutual interference between the cell walls of the 3D-RH structure under quasi-static compression loading, the relationship between the dimensions of each cell wall is specified as follows to ensure that the two horizontal cell walls inside the unit cell do not interfere with each other during the compression process. As shown in Figure 2c, point I and point J do not interfere with each other, and we have $l_{1}\geq 2l_{2}+b$. Also, point D and point A’ do not interfere with each other, and we have $l_{3}\geq l_{2}$.

As for the equivalent compressive strength, all cell walls remain stationary without deformation at the initial moment. Therefore, the initial height along the compression direction, i.e., the distance between points B and H, can be expressed as $H_{0}=2l_{2}\sin\theta$. During the subsequent deformation process, the horizontal cell walls continuously move downwards, and relative rotation occurs between the cell walls. In addition, the connecting cell wall not only moves along the compression direction, but also shrinks laterally towards the center of the cell, and the re-entrant angle continuously decreases, ultimately leading to the densification stage of the 3D-RH structure (see Figure 2d). At this point, the re-entrant angle is approximately 0°, and the distance $H_{f}$ between points B and H along the compression direction is as follows:

(7) $$H_{f}=4t$$

Thus, the effective compression displacement of the structure is as follows:

(8) $$u_{z}=H_{0}-H_{f}=2l_{2}\sin\theta -4t$$

During the deformation process, the external work W is mainly converted into kinetic energy $E_{k}$ and plastic potential energy $E_{p}$. Specifically, under quasi-static compression conditions, kinetic energy can be neglected. Thus, we have the following:

(9) $$W=E_{p}$$

where the external work $W=\sigma_{sT}u_{z}(2bl_{1}-b^{2})$, in which $\sigma_{sT}$ is the theoretical solution for the equivalent compressive strength of the 3D-RH structure.

During the plastic deformation process, the plastic potential energy of the structure is stored in the plastic hinges formed at the intersection of the cell walls, with a total of 16 plastic hinges formed by 8 oblique cell walls. The plastic potential energy is as follows:

(10) $$E_{p}=16M_{p}\theta$$

where $M_{p}=bt^{2}\sigma_{ys}/4$ is the plastic bending moment formed in the oblique cell wall.

Finally, substituting Equations (8) and (10) into Equation (9), the theoretical solution for the quasi-static compressive strength $\sigma_{sT}$ of the 3D-RH structure can be expressed as follows:

(11) $$\sigma_{sT}=\frac{2\sigma_{ys}\theta t}{\left(l_{2}\sin\theta -2t)(2l_{1}-b\right)}$$

where $\sigma_{ys}$ denotes the yield strength of the parent material.

### 2.3. Computational Model

**Figure 3:**
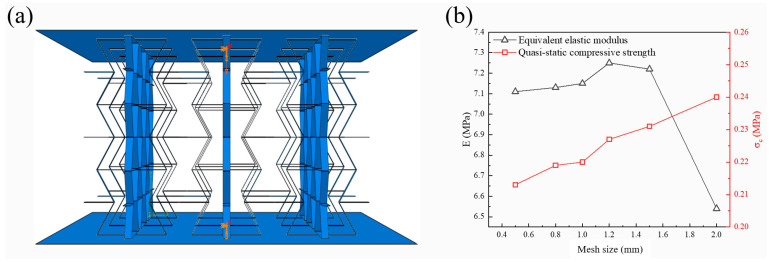
(**a**) Quasi-static and dynamic compression finite element model; (**b**) effect of mesh size on the compression modulus and plateau stress.

To analyze the static and dynamic compressive behavior of the 3D-RH pattern, commercial finite element software Abaqus/Explicit 6.14 is adopted for numerical simulation. As shown in Figure 3a, the middle part is the 3D-RH model, and the upper and bottom plates are rigid plates used to simulate compression conditions. Reference points are set at the center point of the rigid plates to apply loads or boundary conditions. The lower reference point is fixed to constrain the translation and rotation of the lower panel, and a vertical downward load is applied on the upper reference point to simulate the loading process. Displacement load is adopted for the quasi-static condition, and constant velocity load is adopted for the dynamic condition. To prevent the sliding of the structural model, a penalty friction contact with a coefficient of 0.1 is set between the rigid plates and the target structure. In addition, a self-contact is also applied on the middle shell structure itself. The material used for simulation is Al6061, and an isotropic ideal elastoplastic material model is adopted to simulate its quasi-static and dynamic compression mechanical behavior. The density of Al6061 is 2.7 g/cm^3^, the elastic modulus is 40.3 GPa, the initial yield strength is 113.7 MPa, and the Poisson’s ratio is 0.33. All these material parameters are directly provided by the manufacturer [28].

Since all the faces of the 3D-RH configuration are thin shells, the S4R explicit linear shell element is used for mesh generation. The mesh size has a significant effect on the simulation results, and the number and quality of the mesh directly affect the accuracy of the results and the computational scale. Generally, the calculation accuracy will improve as the mesh number increases. When the mesh number increases to a certain extent, the calculation results will gradually converge, but the calculation scale and time will also increase significantly. Therefore, in order to achieve a high computational accuracy and appropriate computational scale, it is necessary to conduct mesh sensitivity analysis. Figure 3b shows the effect of mesh size on the equivalent elastic modulus and compressive strength. It can be seen that, as the mesh size decreases, the equivalent elastic modulus and compressive strength values tend to stabilize and gradually converge. When the mesh size is 1 mm, the error caused by mesh accuracy is within an acceptable range, and the calculation time is not too long. Therefore, the mesh size is determined as 1 mm.

## 3. Validation of Numerical and Theoretical Results

In this section, the finite element results are compared with the experimental ones, and the accuracy of the theoretical results are also validated.

### 3.1. Validation of Finite Element Model

**Figure 4:**
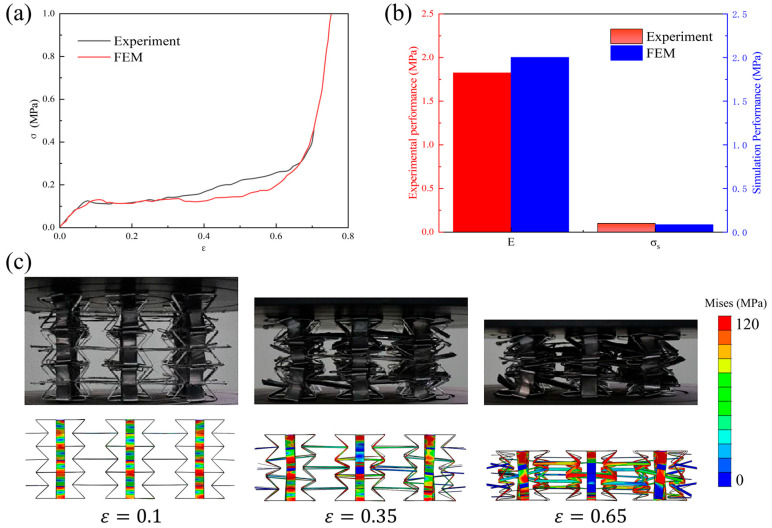
Comparison of the quasi-static compression (**a**) stress–strain curves, (**b**) performance parameters, and (**c**) deformation processes between experiment and simulation.

In this section, finite element analysis results are compared with the experimental ones. The experiment was performed on the Instron universal testing machine. The loading rate was 0.001/s. The stress and strain values were obtained from the experimental force and displacement values. Herein, quasi-static compression stress–strain curves and key performance parameters, as well as the deformation processes, are all listed (see Figure 4). It can be seen that the simulated stress–strain curves match well with the experimental ones, including the initial elastic stage, intermediate plateau stage, and final densification stage. The simulated compressive modulus and plateau stress are 1.985 MPa and 0.094 MPa, respectively. The experimental compressive modulus and plateau stress are 1.878 MPa and 0.102 MPa, respectively. The maximum error between the simulation and the experiment is only 5.39%, demonstrating the accuracy of finite element results. Deformation processes are also plotted and compared in Figure 4c, where the simulated process is consistent with the experimental one, further demonstrating the reliability of the finite element model.

### 3.2. Validation of Theoretical Model

**Figure 5:**
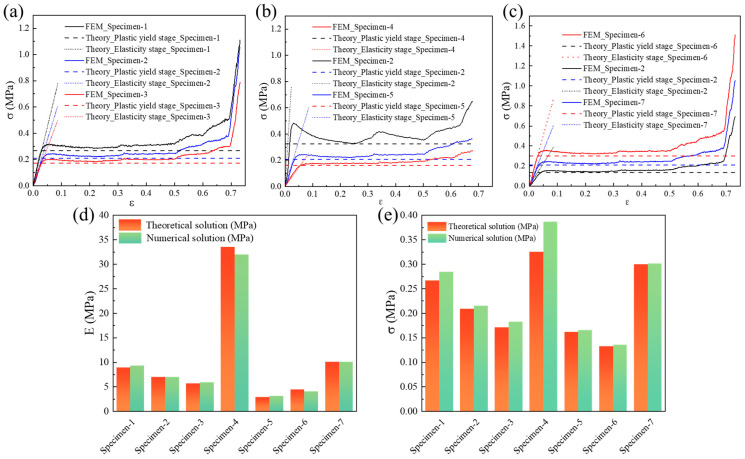
(**a**–**c**) Comparison of the quasi-static compression stress–strain curves between theoretical prediction and numerical simulation from Specimen-1 to Specimen-7; (**d**) comparison of compressive modulus; (**e**) comparison of plateau stress.

After verifying the accuracy of finite element results in the previous section, this section will further validate the quasi-static compressive modulus and compressive strength obtained from the theory analysis. The quasi-static compression stress–strain curves of the Specimen-1 to Specimen-7 patterns from theoretical prediction and numerical simulation are plotted in Figure 5a–c. Herein, stress is obtained by dividing the reaction force by the initial equivalent area, while strain is obtained by dividing the displacement by the initial height. The theoretically predicted curves include the equivalent modulus and platform stress curves, which have a good consistency with the numerical curves. Furthermore, the compressive modulus and plateau stress values from the theory and simulation are summarized in Figure 5d and Figure 5e, respectively. Under a quasi-static loading condition, the maximum errors in the compressive modulus and plateau stress between the theory and simulation are only 8.75% and 8.96%, respectively, demonstrating the accuracy of theory analysis results.

## 4. Results and Discussion

In this section, the quasi-static and dynamic compression mechanical behaviors of the 3D-RH patterns are illustrated, and the corresponding Young’s modulus and compressive strength are both compared with the competing configurations in the Ashby plots. Herein, only the performance curve of Specimen-2 is investigated.

### 4.1. Quasi-Static Compression Behavior

#### 4.1.1. Mechanical Behavior

**Figure 6:**
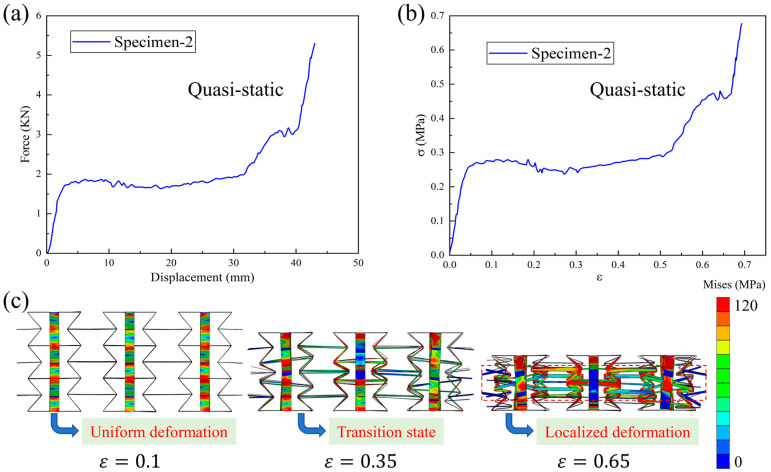
Quasi-static compression (**a**) force–displacement curve, (**b**) stress–strain curve, and (**c**) deformation process of Specimen-2.

Figure 6a,b show the quasi-static compression force–displacement curve and stress–strain curve of Specimen-2, respectively. The typical performance characteristics of cellular materials can be observed: an initial elastic deformation region, intermediate plateau stress segment, and final densification stage. Moreover, one should note that a relatively stable curve can be harvested in Specimen-2, indicating a typical bending-dominated deformation behavior. The corresponding quasi-static compression deformation process is plotted in Figure 6c. When the compression strain is 0.1, a uniform deformation is observed in the structural pattern. At a strain of 0.35, a transition state occurs. Finally, as the compression strain continues to increase, localized deformation occurs in the structure, and the specimen enters the densification stage. For the other structural patterns, similar deformation phenomena can also be observed.

#### 4.1.2. Effect of Geometric Parameter

**Figure 7:**
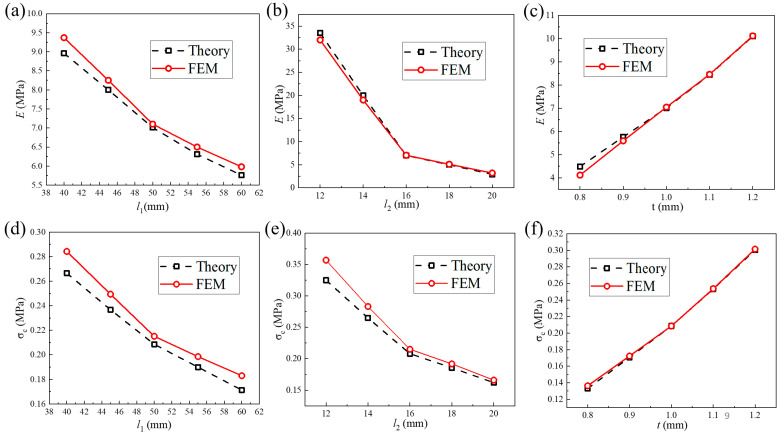
Effect of (**a**) horizontal cell wall length, (**b**) oblique cell wall length, and (**c**) cell wall thickness on the compression modulus. Effect of (**d**) horizontal cell wall length, (**e**) oblique cell wall length, and (**f**) cell wall thickness on the plateau stress.

Herein, the effects of geometric parameters, including the horizontal cell wall length, oblique cell wall length, and cell wall thickness, on the quasi-static compression modulus and plateau stress are illustrated. In Figure 7, E represents the compression modulus of the structural pattern, and $\sigma_{c}$ denotes the plateau stress of the structural pattern. They can be obtained from the compression stress–strain curve of the structural pattern and are two important quantitative indicators for characterizing the performance characteristics of the structures. It can be observed that the quasi-static compression modulus and plateau stress both exhibit a decreasing trend as the horizontal cell wall length or oblique cell wall length increases. When horizontal cell wall length increases from 40 mm to 60 mm, the quasi-static compression modulus will decrease from 9.37 MPa to 5.98 MPa, and plateau stress will decrease from 0.284 MPa to 0.183 MPa. When the oblique cell wall length increases from 12 mm to 20 mm, the quasi-static compression modulus will decrease from 32.0 MPa to 3.20 MPa, and plateau stress will decrease from 0.357 MPa to 0.166 MPa. However, they will increase with the increase in cell wall thickness. When cell wall thickness increases from 0.8 mm to 1.2 mm, the quasi-static compression modulus will increase from 4.12 MPa to 10.1 MPa, and plateau stress will increase from 0.136 MPa to 0.301 MPa.

### 4.2. Dynamic Compression Behavior

The dynamic compression behavior of the novel 3D-RH structure is unclear, which deserves more attention. In this section, three different loading velocities, i.e., 10 m/s, 25 m/s, and 50 m/s, are considered. These three speeds correspond to low-speed impact, medium-speed impact, and high-speed impact, respectively. Through a dynamic compression investigation, the influence of the microstructure effect on the dynamic properties can be revealed.

#### 4.2.1. Effect of Loading Velocity

**Figure 8:**
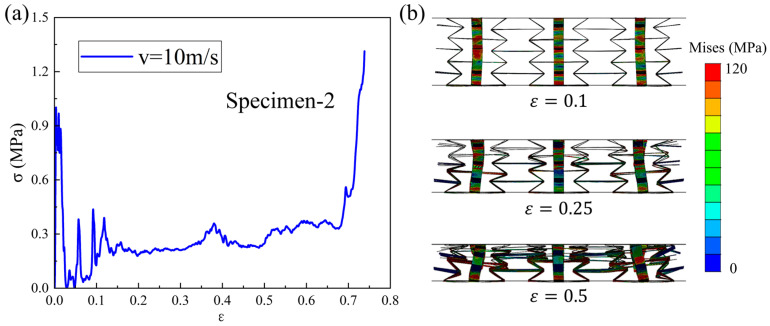
Dynamic compression (**a**) stress–strain curve and (**b**) deformation process of Specimen-2 under low-speed loading condition.

Figure 8a shows the dynamic compression stress–strain curve of the Specimen-2 pattern under a 10 m/s loading velocity. It can be seen that the performance curve exhibits an initial peak stress followed by an oscillating plateau stress and a rapid rising stress, which displays the typical performance characteristics of cellular materials under dynamic loading conditions. When compared with the mechanical performance under quasi-static loading conditions, an improvement can be seen in the macroscopic mechanical response of the structure under low-speed loading. The corresponding compression process is plotted in Figure 8b. The 3D-RH structure continues to contract inward during the compression process, and the deformation degree of each layer cell is slightly different, with the deformation degree of the upper and middle cells being more significant. The deformation mode of the cells in each layer of the structure is the same, mainly formed by plastic hinges at the intersection of the cell walls. At a compression strain of 0.1, the deformation degree of each layer is similar. When strain is 0.25, the deformation of the upper and middle cells becomes more pronounced and the overall structure shrinks inward, exhibiting a significant negative Poisson’s ratio effect. As the strain continues to increase to 0.5, each layer of cells gradually collapses. At this point, the upper and middle layers of cells have already collapsed, while the lower layer of cells is still in the compression process.

**Figure 9:**
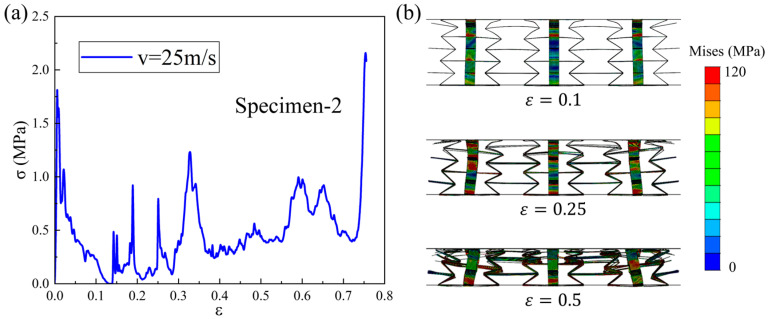
Dynamic compression (**a**) stress–strain curve and (**b**) deformation process of Specimen-2 under medium-speed loading condition.

The dynamic compression stress–strain curve of the Specimen-2 pattern under a 25 m/s loading velocity is plotted in Figure 9a. Compared with the stress–strain curve under low-speed loading, a performance improvement is also obtained under medium-speed loading, but the oscillation is also more severe. As can be seen from Figure 9b, the deformation of the Specimen-2 pattern undergoes significant changes under the medium-speed loading condition. The deformation of the upper layer cells includes plastic hinges at the intersection of cell walls and the bending deformation of some oblique cell walls, while the deformation of the middle and lower layers of cells is still dominated by plastic hinges [29]. During the compression process, the deformation of the upper cell is more significant, and its oblique cell wall undergoes a significant bending deformation. This is because the impact force of the loading plate is greater at this speed, and the deformation degree of the upper cell is greater. Thus, the bending stiffness of the structural oblique cell wall is not sufficient to withstand the impact force during rotation, resulting in bending deformation. When the compression strain is 0.1, the deformation of the upper cell is more obvious, and some oblique cell walls undergo bending deformation. When the strain is 0.25, the deformation of the upper cell is significant, and some oblique cell walls are almost pressed to the horizontal, with the re-entrant angle pressed to zero degrees. As the strain continues to increase to 0.5, the upper and middle cells have already collapsed, while the lower cells are still in the compression process.

**Figure 10:**
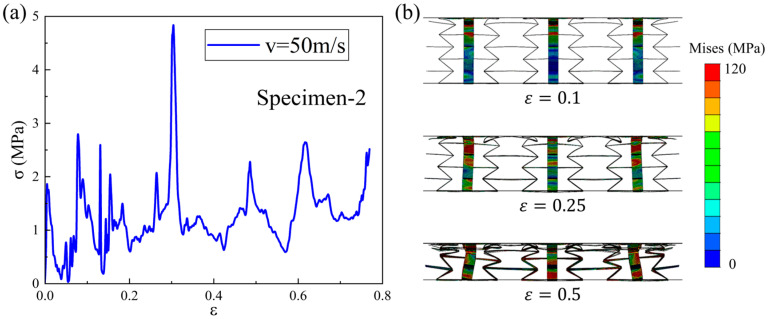
Dynamic compression (**a**) stress–strain curve and (**b**) deformation process of Specimen-2 under high-speed loading condition.

In Figure 10a, the dynamic compression stress–strain curve of the Specimen-2 pattern under a 50 m/s loading velocity is plotted. Compared with those under the low-speed or medium-speed loading, some improvement, coupled with a greater oscillation, can also be observed, which is closely related to the deformation process shown in Figure 10b. Under high-speed impact conditions, structural deformation is mainly dominated by the bending deformation of oblique cell walls. At this point, the impact force generated by the upper plate far exceeds the limit that the structure can withstand. At the moment of contact, the upper layer of the structure undergoes drastic changes, while the deformation of the middle and lower layers is little. Thus, the differences in deformation between each layer are significant. When the compression strain is 0.1, the deformation at the contact position with the upper plate is severe, while the deformation in the remaining parts is not significant. When the compression strain is 0.25, the upper half of the upper cell has been crushed, while the compression deformation of the middle and lower layers is still not significant. As the loading continues, each layer of cells exhibits a phenomenon of layer-by-layer collapse. For example, at a strain of 0.5, the upper cells have been compacted, the middle cells are almost compacted, and the lower cells are still in the compression stage.

#### 4.2.2. Parametric Analysis

**Figure 11:**
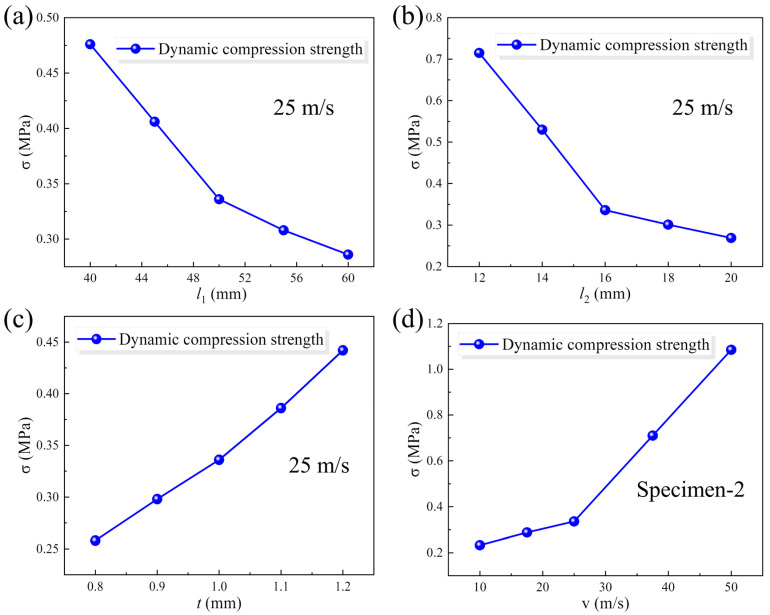
Effect of (**a**) horizontal cell wall length, (**b**) oblique cell wall length, and (**c**) cell wall thickness on the plateau stress under medium-speed loading condition. (**d**) Effect of loading velocity on the dynamic compression plateau stress.

Dynamic compression strength, i.e., plateau stress, is an important indicator in characterizing the impact resistance characteristics of structural materials. The plateau stress in the dynamic analysis is calculated as the average stress before a strain of 0.6. Herein, the effects of the horizontal cell wall length, oblique cell wall length, and cell wall thickness on the plateau stress under medium-speed loading conditions are illustrated in Figure 11a–c, respectively. The dynamic compression strength exhibits a decreasing trend as the horizontal cell wall length or oblique cell wall length increases, while it increases with the increase in cell wall thickness. The dynamic compression strengths of Specimen-1 to Specimen-7 are, respectively, 0.476 MPa, 0.336 MPa, 0.286 MPa, 0.715 MPa, 0.269 MPa, 0.258 MPa, and 0.442 MPa under medium-speed loading conditions. In addition, the dynamic compression plateau stress values under different loading velocities are given in Figure 11d. When the loading velocity increases from 10 m/s to 50 m/s, the dynamic compression plateau stress can increase from 0.233 MPa to 1.085 MPa. A tremendous growth rate of 366% can be achieved, indicating an apparent strain rate effect in the dynamic mechanical properties of the 3D-RH structural pattern.

### 4.3. Comparison with Competing Configurations

**Figure 12:**
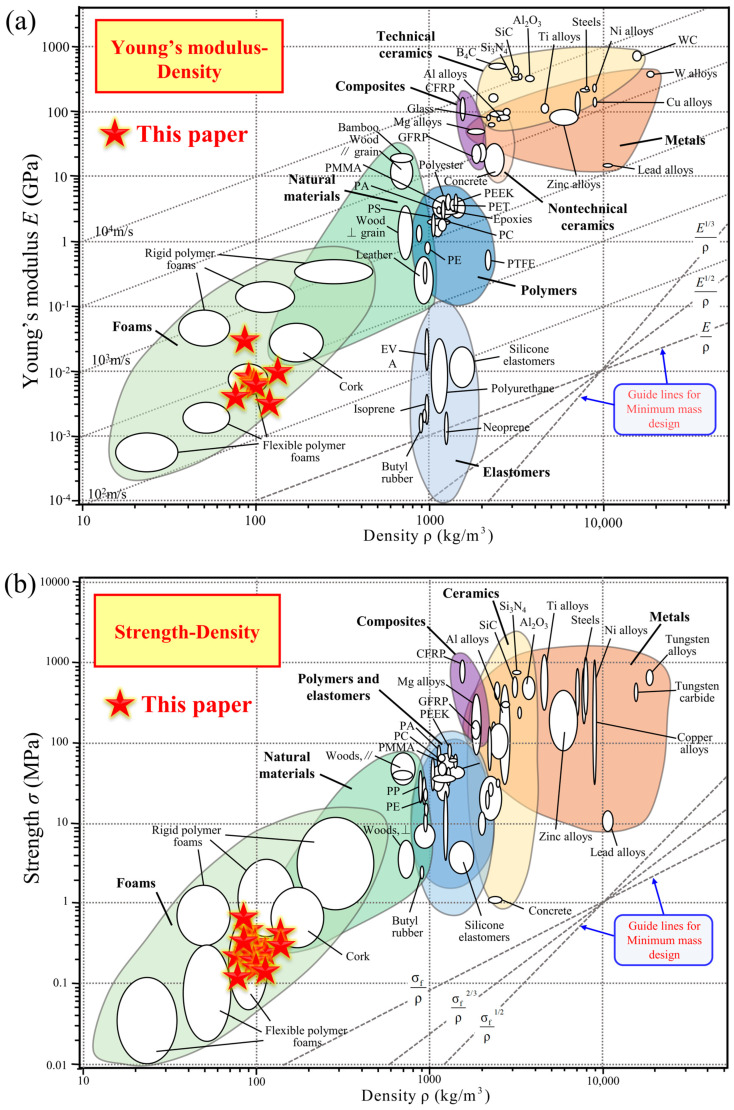
Comparison of the (**a**) Young’s modulus and (**b**) compressive strength of the 3D-RH structural patterns, with competing configurations.

Based on the simulated compressive modulus and compressive strength of the 3D-RH structural patterns, Ashby maps are plotted in Figure 12. The horizontal axis represents the mass density, and the vertical axis represents the key performance parameter (Young’s modulus or strength). In addition, for a more intuitive comparison, the performance parameters of common structural materials, including elastomers, foams, natural materials, polymers, composites, nontechnical ceramics, metals, and technical ceramics, are all marked. They are some common materials in engineering. Our goal is to achieve a high stiffness and strength while pursuing a lightweight design. In the upper-left corner of the two figures below, the mass density is relatively low while the performance indicators are strong, which is an ideal area. It can be seen that, from the perspective of the innovative development of lightweight, high-specific-stiffness, and high-specific-strength structural materials, the 3D-RH structure proposed in this paper has acceptable mechanical properties (i.e., compressive modulus and compressive strength), and is within the region of foams, demonstrating potential attractive application prospects.

## 5. Conclusions

Inspired by the deformation mechanism of the traditional two-dimensional re-entrant honeycomb (2D-RH) structure, a novel three-dimensional re-entrant honeycomb (3D-RH) structure is constructed by expanding the dimensions of the two-dimensional structure. A series of methods including experimental testing, theoretical analysis, and numerical simulation are all utilized to study its quasi-static and dynamic compressive mechanical properties and deformation processes. The main conclusions are summarized as follows:

(1) The quasi-static compressive performance curve of the 3D-RH pattern is a typical bending-dominated deformation behavior. The typical performance characteristics of cellular materials can be observed: an initial elastic deformation region, intermediate plateau stress segment, and final densification stage. The corresponding compression modulus and plateau stress both exhibit a decreasing trend as the horizontal cell wall length or oblique cell wall length increases. However, they will increase with the increase in cell wall thickness.
(2) The dynamic compressive performance curve of the 3D-RH pattern exhibits an initial peak stress followed by an oscillating plateau stress and a rapid rising stress. The dynamic compression strength exhibits a decreasing trend as the horizontal cell wall length or oblique cell wall length increases, while it increases with the increase in cell wall thickness. In addition, the dynamic mechanical properties of the 3D-RH structural pattern exhibit an apparent strain rate effect. When the loading velocity increases from 10 m/s to 50 m/s, the dynamic compression plateau stress can increase from 0.233 MPa to 1.085 MPa.
(3) Based on the performance comparison in the Ashby maps, the 3D-RH structure proposed in this paper has an acceptable quasi-static and dynamic compressive modulus and compressive strength, and is within the region of foams, demonstrating its potential attractive application prospects in the innovative development of lightweight, high-specific-stiffness, and high-specific-strength structural materials.

## Figures and Tables

**Table 1:** All the geometric parameter values of the 3D-RH structure. Herein, the most important structural characteristic of a cellular material is its relative density, which is defined by the ratio of the density of a cellular material to the density of constituent material.

Specimen	$l_{1}$ (mm)	$l_{2}$ (mm)	$l_{3}$ (mm)	t (mm)	θ (°)	b (mm)	Relative Density
Specimen-1	40	16	27.87	1	36.42	8	0.033
Specimen-2	50	16	22.87	1	36.42		0.035
Specimen-3	60	16	17.87	1	36.42		0.036
Specimen-4	50	12	17.33	1	52.34		0.030
Specimen-5	50	20	27.60	1	28.36		0.039
Specimen-6	50	16	22.80	0.8	36.87		0.027
Specimen-7	50	16	22.95	1.2	35.98		0.042

## Data Availability

The original contributions presented in this study are included in the article. Further inquiries can be directed to the corresponding authors.
